# Tuberculosis Exhibit at Washington

**Published:** 1908-10

**Authors:** 


### BUFFALO MEDICAL JOURNAL.

**A Monthly Review of Medicine and Surgery.**

*EDITOR:*

WILLIAM WARREN POTTER, M. D.

All communications, whether of a literary or business nature, books for reviewand exchanges, should be addressed to the editor 238 DELAWARE AVE., BUFFALO, N. Y

VOL. LXIV. OCTOBER, 1908. No. 3

### Tuberculosis Exhibit at Washington.

WE have dealt with the Congress itself heretofore in these columns; therefore, in this article we shall handle briefly the antituberculosis museum connected with the Sixth International Congress for combating tuberculosis now in session at Washington. In the first place it is interesting to observe that almost every civilised country is represented in this wonderful museum, and nearly every state of this republic presents evidence of what it is doing to prevent or cure pulmonary consumption. A glance through the various rooms is sufficient to indicate that the prevailing idea, the underlying thought of prevention, is fresh air day and night, everywhere fresh air, sleeping or waking, and that fresh air is even more important, more necessary during the night time than in the day time. The devices for protection from inclement weather while the person is living in the open air are indeed many, and are such as to fit almost every purse. The poor and the rich alike are provided with whatever is needed to afford protection from high winds or low temperatures, according to locality and the stages of the malady that is being combated.

The exhibit, therefore, embraces a most interesting array of tents, shacks, cottages, and hospitals, some of which are full size and others being models, the latter applying to hospitals and cottages in particular. One tent, which appears to be about the size of the army hospital tent, say 14 feet by 16 feet, fitted up with necessary conveniences, in our view presented a most attractive appearance, and is the one we should choose for ourselves should it become necessary. But there is such a great variety of these "habitations" that all conditions as to cost and preference are completely provided for. Hospitals and sanitaria in every conceivable form and size, together with surrounding grounds are exhibited in models, the wonder being that all these various miniature creations should find place in the museum.

It is here that all the important questions relating to the construction of new hospitals may be determined, new ideas obtained and the latest schemes in ventilation, drainage, lighting, heating, as well as the multifarious minor problems relating to buildings and grounds may be discussed and evolved. We were impressed with a bird's eye view of Switzerland, giving its mountains, valleys, lakes and rivers in relief, in the midst of which was the government sanitarium for consumptives. The entire German exhibit is attractive and its completeness is a subject of common remark.

The United States must not be overlooked in estimating the usefulness of the exhibit. The general government need not be ashamed of its display, nor, indeed, should such of the states as are making exhibits feel anything but pride in them. One of the striking exhibits is that of three models of tenement houses, made by the committee on the prevention of tuberculosis of the charity organisation society of the City of New York. The first is a block of tenements on the east side in which 2,781 persons live in 1,588 rooms without one bath and but 264 water closets. Only 40 apartments are supplied with hot water. There are 441 dark rooms, having no ventilation to the outer air and no light or air except that derived from other rooms, and 635 rooms getting their sole light and air from dark and narrow airshafts. During five years there were recorded 32 cases of tuberculosis from this block, and during one year 13 cases of diphtheria. In addition, during five years, there were 665 different applications for charitable relief. The gross rentals amount to $113,964.00 a year. This block is not one of the worst in the city.

The second is a typical block of "dumb-bell" tenements erected in accordance with laws in force prior to 1901. The halls and 10 out of 14 rooms on each floor are dark and ill-ventilated, receiving their sole light and air from the narrow airshafts that give almost no light below the top floors. The third is a block built in accordance with the new law and is about as complete as modern methods of sanitation can make it. There is much food for thought here on the subject of tenement house construction.

There is much of interest and instruction in this museum; indeed, it is full of the most instructive object lessons regarding the prevention and cure of this most destructive malady, one that causes the death of nearly 15,000 persons annually in the State of New York alone, and this in spite of all the empire state is doing in the way of prevention and cure. There remains much more to do. Let all who can do so visit this wonderful exhibit and take new inspiration, new courage, for the continuation of the warfare in the interest of the prevention as well as cure of tuberculosis.

